# An injectable self-adaptive polymer as a drug carrier for the treatment of nontraumatic early-stage osteonecrosis of the femoral head

**DOI:** 10.1038/s41413-022-00196-y

**Published:** 2022-03-12

**Authors:** Ning Kong, Hang Yang, Run Tian, Guanzhi Liu, Yiyang Li, Huanshuai Guan, Qilu Wei, Xueshan Du, Yutian Lei, Zhe Li, Ruomu Cao, Yiwei Zhao, Xiaohui Wang, Kunzheng Wang, Pei Yang

**Affiliations:** 1 Department of Bone and Joint Surgery, Second Affiliated Hospital of Xi’an Jiaotong University, No. 157 Xiwu Road, Xi’an, 710004 China; 2 Harvard John A. Paulson School of Engineering and Applied Sciences, Harvard University, Cambridge, MA 02138 USA; 3 Department of Dermatology, Second Affiliated Hospital of Xi’an Jiaotong University, No. 157 Xiwu Road, Xi’an, 710004 China; 4 Department of Spine Surgery, Honghui Hospital of Xi’an Jiaotong University, No. 555 Youyi East Road, Xi’an, 710000 China

**Keywords:** Bone, Bone quality and biomechanics

## Abstract

Core decompression (CD) with the elimination of osteonecrotic bone is the most common strategy for treating early-stage nontraumatic osteonecrosis of the femoral head (ONFH). Adjuvant treatments are widely used in combination with CD as suitable methods of therapy. Existing augmentations have to be fabricated in advance. Here, we report a novel injectable glycerin-modified polycaprolactone (GPCL) that can adapt to the shape of the CD cavity. GPCL shows great flowability at 52.6 °C. After solidification, its compressive modulus was 120 kPa at body temperature (37 °C). This excellent characteristic enables the polymer to provide mechanical support in vivo. In addition, GPCL acts as a carrier of the therapeutic agent zoledronic acid (ZA), demonstrating sustained release into the CD region. ZA-loaded GPCL was injected into ONFH lesions to treat early-stage nontraumatic cases. Compared to that in the CD group, CD+ZA-loaded GPCL injection preserved bone density and increased the collagen level in the femoral head. At the interface between the GPCL and CD tunnel wall, osteogenesis was significantly promoted. In addition, morphological evaluations revealed that the femoral heads in the CD+ZA-GPCL group exhibited improved pressure resistance. These results suggest a strategy effective to preserve the bone density of the femoral head, thus decreasing the possibility of femoral head collapse. This novel injectable polymer has, therefore, considerable potential in clinical applications.

## Introduction

Osteonecrosis of the femoral head (ONFH) is a devastating disease that causes extreme pain and disability. This condition is bilateral in more than 60% of patients.^1^ Those suffering from this condition are mostly young or middle aged.^2^ In the US, more than 20 000 individuals are afflicted by ONFH annually, the prevalence of which continues to increase.^3,4^ In China, it has been estimated that more than 8 million individuals cumulatively have suffered from ONFH.^5^ The incidence of ONFH has been reported to be 1.4 per 100 000 in the UK, which is close to that reported for Japan (1.9 per 100 000).^6^ ONFH patients suffer from both physical discomfort and severe mental pressure during social activities.^7^ Glucocorticoid-associated ONFH (GA-ONFH) is predominant in nearly half of all ONFH cases.^2^ Regardless of the cause, more than 80% of femoral heads will collapse if patients receive no intervention.^8^ Following collapse, total hip arthroplasty (THA) remains the only treatment strategy that can relieve pain and restore joint functionality. The mean cost for THA surgery is approximately $23 650,^9^ which is a substantial financial burden for the majority of patients. Although surgical methods and material science for THA have been developed, complications continue to occur. For example, minimally invasive surgical approaches and advanced prostheses have dramatically optimized surgical outcomes, but complications such as periprosthetic infection and aseptic loosening remain severe challenges.^10^ In addition, due to the limited lifespan of prostheses, approximately 40% of patients under 35 years of age will have to undergo revision surgery 20–25 years after primary THA.^11^ Given these difficulties, femoral head collapse must be prevented to delay or even avoid THA.

A series of treatment strategies have been used in the precollapse stage of ONFH.^12^ Core decompression (CD) is the most commonly used therapy and exhibits superior outcomes.^4,13^ However, in early-stage ONFH, simple CD has been shown to exhibit a 29%–52% rate of failure in various clinical trials.^14^ Furthermore, approximately 38% of ONFH patients underwent THA with a mean interval of 26 months after simple CD.^15^ Although certain late-stage ONFH patients were included in the trials, the intrinsic disadvantages of CD increase the possibility of failure because the procedure theoretically weakens local tissue, resulting in associated fractures or even additional collapse of the femoral head.^16^ Adjuvant techniques, such as free vascularized bone grafts, porous tantalum rod implantation, and cell-seeded scaffolds combined with CD, have demonstrated improved efficacy.^17–19^ However, additional studies are required to properly evaluate these approaches.

To date, no medication has been identified as a standard cure for this disease. Anti-osteoporosis medicines, such as bisphosphonates (BPs), may effectively treat GA-ONFH because bone volume loss-induced subchondral fractures initiate femoral head collapse,^20^ and collapse is caused by microfractures in the subchondral bone regardless of the cause. It is generally thought that collapsed regions in ONFH occur in tissue with significantly decreased bone volume fraction, trabecular thickness, and bone mineral density.^21^ Furthermore, the rate of collapse progression is positively correlated with the bone resorptive volume ratio in ONFH patients.^22^ BPs, such as alendronic acid and zoledronic acid, have been approved by the Food and Drug Administration (FDA) to treat osteoporosis.^23^ These agents have also induced beneficial therapeutic outcomes in diverse neoplastic or metabolic bone diseases, such as multiple myeloma and Paget’s disease.^24,25^ However, BPs cause many unavoidable side effects, even though the therapeutic results are encouraging. Oral BPs have low absorption and variable bioavailability depending on the individual. Approximately 99% of orally administered BPs are excreted into the feces unmodified.^26^ Intravenous BPs are likely to be eliminated via the kidneys and thus result in renal impairment, necessitating a strict limitation for patients with poor renal function.^27^ Osteonecrosis of the jaw is a severe side that is effect closely associated with the administration of BPs, with an incidence of 3%–4% in randomized controlled trials.^28,29^ In addition, oral or intravenous administration may not achieve sufficient drug concentrations due to insufficient blood supply in the osteonecrotic region. Hence, local administration of BPs should be considered to improve the utilization rate and reduce systemic side effects.

**Scheme 1:**
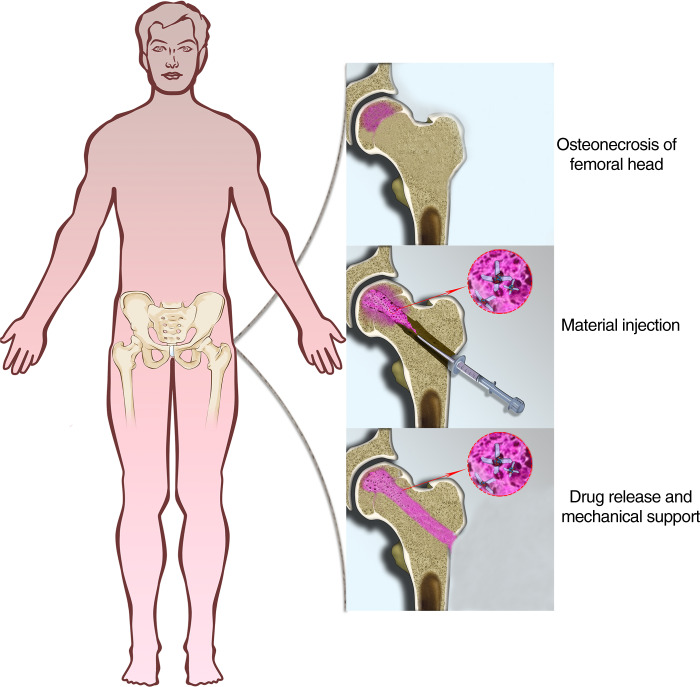
Core decompression combined with injection of ZA-loaded GPCL to treat early-stage osteonecrosis of the femoral head

Studies have explored the effect of biomaterials, such as modified alginate, chitosan, gelatin, collagen, and hyaluronic acid, in combination with CD in treating ONFH.^17,30^ In addition, some artificial polymers, including poly(vinyl alcohol) (PVA), poly(ethylene glycol) (PEG), and poly(lactic-co-glycolic acid) (PLGA), potentially act as drug release systems to assist CD. These polymers help bone regeneration through implantation, in situ polymerization, or injection by grafting the functional group onto the polymer or mix.^31–34^ The application of porous tantalum rods achieved satisfying outcomes in treating precollapse ONFH according to an average 8-year follow-up.^35^ However, multifunctional biomaterials that are injectable and degradable, have sustained release, and are compatible with different drugs are still needed. The previous strategies can only meet part of the requirements. It is still worth exploring the augmentation of CD. In the present study, we report a novel injectable biomaterial as a drug delivery system based on modified poly(ε-caprolactone) (PCL). This biomaterial could provide mechanical support to the CD channel and sustainably release zoledronic acid during biodegradation to treat early-stage GA-ONFH (Scheme 1).

## Results

### Synthesis and characterization

PCL is widely used to fabricate scaffolds, drug carriers, and medical devices due to its excellent biocompatibility and mechanical properties.^36^ It has also been approved by the FDA and is currently used in clinics. PCL is a biodegradable material and is favorable for use in vivo because it obviates the need for additional surgery for removal. The degradation rate of PCL depends on its molecular weight, polymer crystallinity, and environmental conditions,^37^ and it usually takes months to years to degrade completely. The degradation of PCL is autocatalyzed by the carboxylic acid group that is liberated during hydrolysis, in addition to the action of many enzymes, resulting in more rapid synergistic decomposition. PCL can be fabricated into printed structures, nanofibers, and nanoparticles for bone tissue engineering.^38–40^ However, prefabricated structures often do not fit in the complex shapes of lesions. Injectable materials represent the most convenient biomaterial for fabrication and implantation. However, the melting temperature of medical polymers such as PCL is usually higher than 60 °C, and they have high viscosity, which prevents them from being injectable. Such high melting temperatures may deactivate thermolabile drugs, such as proteins and peptides, and cause damage to osteocytes.

**Fig. 1:**
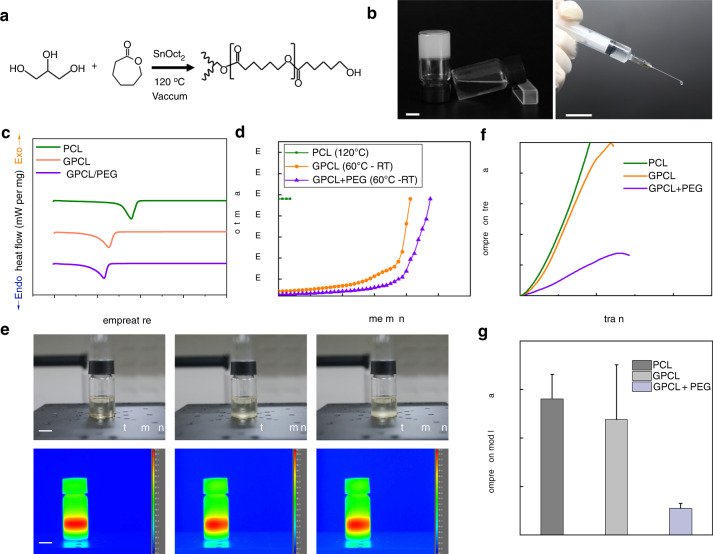
Synthesis and characterization of GPCL. **a** Synthesis of GPCL. **b** The polymer was a liquid when cooled to room temperature. **c** Melting temperatures of GPCL polymers, as measured by DSC. **d** The viscosity of the polymer during cooling from 60 °C. **e** Thermal and optical images of GPCL/PEG cooling from 60 °C to room temperature. **f**, **g** Compressive strength and modulus of the polymers at 37 °C. Scale bars: 1 cm

In the present study, we proposed the formulation of a modified injectable polymer based on PCL. This material consisted only of the biocompatible materials glycerin and PCL. Glycerin-modified PCL (GPCL) was synthesized by mixing glycerin with PCL at a certain ratio of monomers through ring-opening polymerization in a catalyzed reaction (Fig. 1a and S1). The GPCL was liquid when warmed slightly to ~60 °C and was therefore able to be injected but was solid at physiological temperature (37 °C) (Fig. 1b). Differential scanning calorimetry (DSC) measurements indicated that unmodified PCL had a melting point of 57.8 °C, while the melting point was 52.6 °C for GPCL (Fig. 1c). To further control the duration of degradation, PEG was added to GPCL, causing the melting point to decrease slightly to 51.5 °C (Fig. 1c). Although the melting temperature of unmodified PCL was only 5.2 °C higher than that of GPCL, the viscosity of unmodified PCL remained at 1.9 × 10^4^ mPa‧s even at 120 °C, which was too viscous to be injectable (Fig. 1d). GPCL melted at just 60 °C, which was a relatively benign temperature, but maintained its liquid state for more than 10 min when cooled to room temperature (25 °C) (Fig. 1d), and GPCL had a viscosity of less than 3.5 × 10^3^ mPa‧s, which was consistent with smooth injection. Furthermore, the addition of PEG also decreased the viscosity of the polymer. The thermal analysis results showed that GPCL/PEG maintained its liquid state even when the temperature was less than the melting point during cooling (Fig. 1e). The long duration at a low viscosity would enable clinicians to inject the material or mix drugs. After sufficient cooling (~20 min) and solidification of GPCL, its compressive modulus was 120 kPa at body temperature (37 °C), which was comparable to that of unmodified PCL (140 kPa) (Fig. 1f–g). GPCL/PEG had a modulus of 8 kPa; therefore, the modulus of the polymer could be matched to that of the bone tissue by adjusting the concentration of PEG without appreciably changing the melting temperature or viscosity. The thermal characteristics of the polymer enabled different drugs (such as antibiotics or hydroxyapatite) to be mixed into the polymer (Fig. S2) before injection into the bone lesion (Fig. S3).

### Biocompatibility, biodegradability, and rate of drug release

**Fig. 2:**
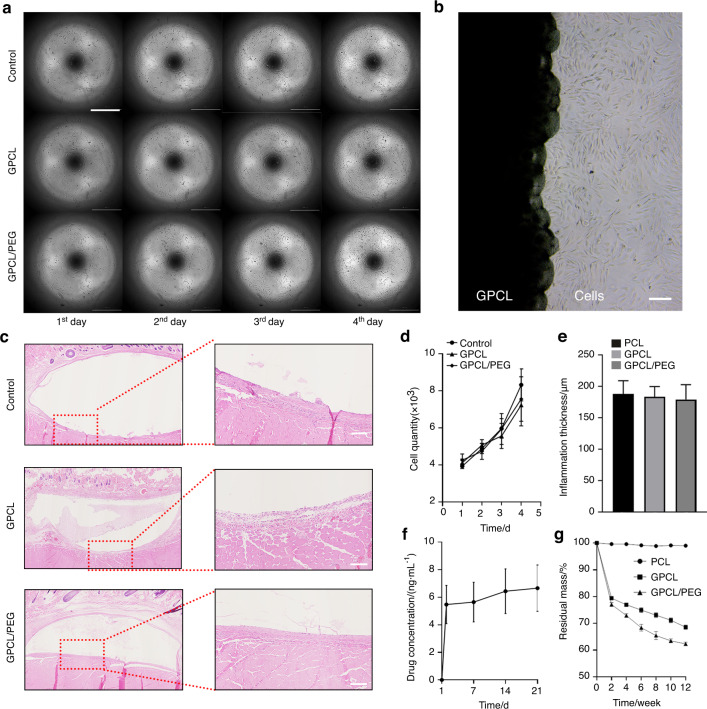
Biocompatibility and drug release from GPCL. **a** The proliferation of MC3T3-E1 cells in material extracts. Scale bar: 1 mm. **b** MC3T3-E1 cells were cultured with GPCL. Scale bar: 100 μm. **c** Subcutaneous implantation of materials. Scale bar: 100 μm. **d** Quantification of cell numbers in the cell proliferation assays. **e** Quantification of the area of inflammation, as determined by implantation assays. **f** Rate of release of vancomycin loaded into GPCL. **g** The degradation rate of GPCL in vivo. All values represent the mean ± SEM (*n* ≥ 6)

Satisfactory biocompatibility is required for biomaterials to be used in clinical applications. As an implantable biomaterial, GPCL exhibited high levels of biocompatibility in vivo and in vitro. Cell proliferation in culture was measured using a live-cell imaging system (Cytation5, BioTek, USA) for 4 days, and the cell quantity was calculated automatically (Video S1). Images were captured at different time points. The results suggested that MC3T3-E1 cells in the different groups maintained a regular shape and were distributed evenly (Fig. 2a). After 4 days of culture, the cell number increased from approximately 4 100-7 700 per well. The cell numbers in the different groups were not significantly different at these time points (*P*_day1_ = 0.178, *P*_day2_ = 0.428, *P*_day3_ = 0.624, *P*_day4_ = 0.285), indicating that neither GPCL nor its degradation products reduced cell proliferation (Fig. 2d). In addition, sterile GPCL was coated onto 10-cm diameter culture dishes onto which MC3T3-E1 cells were seeded and cultured for 3 days with a complete culture medium. On day 3, MC3T3-E1 cells were observed using an inverted microscope. At the edge of the GPCL, the cells were densely distributed and displayed normal morphology (Fig. 2b). Furthermore, PCL, GPCL, and GPCL/PEG were implanted subcutaneously in female rats for 2 weeks to evaluate biocompatibility in vivo. Histological sections were analyzed and indicated that a thin layer of fibrous tissue had enclosed the implanted biomaterials with only slight inflammatory cell infiltration, resulting from both the reaction to the implants and mechanical friction (Fig. 2c). Analysis of the thickness of the fibrous layer demonstrated that there was no significant difference between the three groups (*P* = 0.613) (Fig. 2e). Furthermore, sections of the different organs (hearts, livers, spleens, lungs, and kidneys) were evaluated, and the results demonstrated that the implanted materials and their degradation products caused no morphologic changes at the organ level (Fig. S4). These findings suggested that GPCL and GPCL/PEG might be safe for clinical applications.

Biodegradability is critical for implantable polymers because they do not require secondary removal surgery, which could cause further damage. To evaluate the biodegradation of these materials, unmodified PCL, GPCL, and GPCL/PEG were fabricated into cylinders (15 mm long, 5 mm diameter). Each sample was then immersed in 10 mL of PBS (pH 7.4) to measure degradation. Each material was weighed six times after freeze-drying for 24 h every 2 weeks. GPCL and GPCL/PEG exhibited relatively faster degradation rates than unmodified PCL, which exhibited approximately 1.02% weight loss over 12 weeks. During this assay, small particles of the material were evident at the ends of the GPCL cylinders, which exhibited 31.47% weight loss after 12 weeks, while a weight loss of 37.71% was observed in GPCL/PEG (Fig. 2g). Therefore, PEG allows the rate of degradation to be adjusted for different medical applications, especially when there is no requirement for mechanical strength. The biodegradability of GPCL renders additional removal surgery unnecessary, allowing additional volume for bone regeneration while offering mechanical support in the CD tract during early-stage ONFH treatment.

To evaluate the rate of drug release, 1 μg of vancomycin was mixed evenly into GPCL before the material was fabricated into cylindrical implants, as described above. The polymer was immersed in 10 mL of PBS (pH 7.4) at 37 °C, and the drug concentration was measured at different time points. The results indicated that vancomycin exhibited burst release on the first day, during which the concentration in the surrounding solution rose from 0 to 5.466 ± 1.388 ng·mL^−^^1^. The release rate then dropped to a steady rate, with 6.652 ± 1.676 ng·mL^−1^ observed after 3 weeks (Fig. 2f). This characteristic allows GPCL to act as a useful drug delivery system, allowing the loading of therapeutic agents at a relatively low temperature and continuous release.

### Therapeutic effect in vitro

**Fig. 3:**
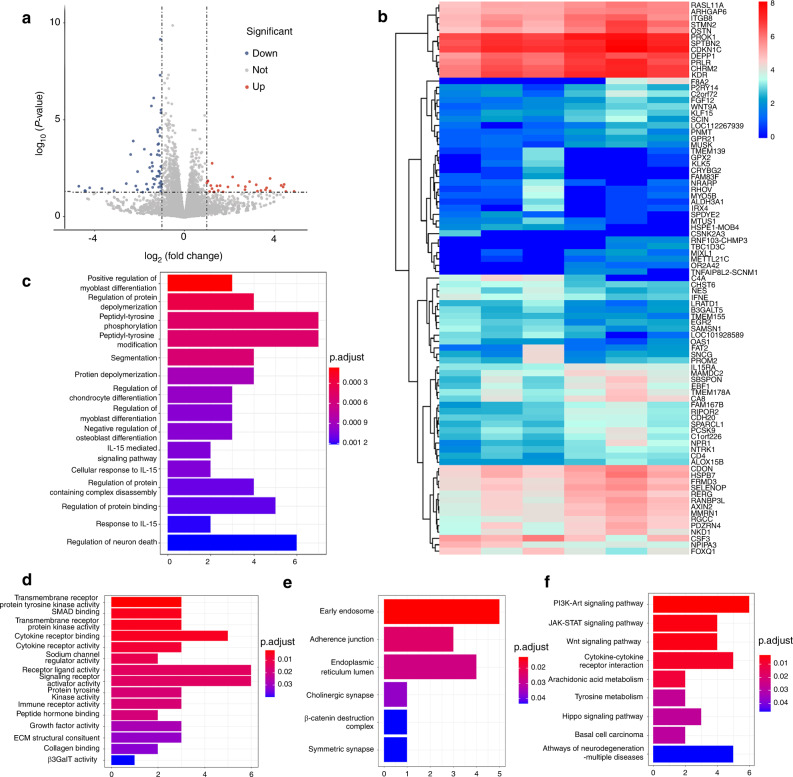
Bioinformatic analysis of the effect of ZA on hMSCs after 14 days in vitro. **a** Volcano plot showing 16 643 genes from the RNA sequencing data. Red points represent upregulated DEGs with log_2_fold change ≥ 1 and *P* < 0.05. Blue points represent downregulated DEGs with log_2_fold change ≤ –1 and *P* < 0.05. **b** Heatmap showing DEG expression levels. **c** GO biological process analysis of DEGs. **d** GO molecular function analysis of DEGs. **e** GO cell component analysis of DEGs. **f** KEGG signaling pathway enrichment analysis of DEGs (*n* = 3)

To explore the effect and potential mechanism of ZA on the proliferation and differentiation of human bone marrow mesenchymal stem cells (hMSCs), the cells were cultured in osteogenic induction medium (OIM) with or without low concentrations of ZA (10^−8^ mol·L^−1^). Generally, low-concentration ZA (10^−8^ mol·L^−1^) administration significantly promoted osteogenesis in vitro, as verified by staining and RT-qPCR after 7 and 14 days of culture (Fig. S5). Furthermore, cells were then collected for mRNA sequencing at the indicated time points. The transcripts from a total of 16 643 genes were sequenced, and 87 differentially expressed genes (DEGs) were identified (*P* < 0.05 and |log_2_fold change|≥1), with 31 genes that were significantly upregulated and 56 genes that were significantly downregulated. A volcano plot and heatmap of the 87 DEGs are shown in Fig. 3a, b. Certain osteoblast-related genes, such as NOTCH-regulated ankyrin repeat protein (NRARP), aldehyde dehydrogenase 3 family member A1 (ALDH3A1), and Multimerin 1 (MMRN1), were significantly differentially expressed. Gene ontology (GO) biological process analysis demonstrated that three osteogenic inhibitory genes (RAN binding protein 3-like (RANBP3L), osteocrin (OSTN), and AXIN2) were significantly downregulated. RANBP3L (log_2_fold change = −1.468 2, *P* = 0.000), OSTN (log_2_fold change = −1.783 3, *P* = 0.000), and AXIN2 (log_2_fold change = −1.145 8, *P* = 0.000) exhibited significantly lower expression in the ZA group than in the control group (Fig. 3b). These three genes were negative regulators of osteoblast differentiation (Fig. 3c). In addition, as demonstrated by GO analysis, some other positive osteogenetic regulatory genes were upregulated and negative osteogenetic regulatory genes were downregulated, and these genes have been shown to affect osteogenesis.^41–43^ Positive osteogenic genes such as NRARP (log_2_fold change = 2.702 3, *P* = 0.037) and ALDH3A1 (log_2_fold change = 3.412 6, *P* = 0.041) were upregulated, while negative MMRN1 (log_2_fold change = −1.183 02, *P* = 0.012) was downregulated. Synergism was observed, in which negative regulatory genes that were downregulated decreased osteoblastic processes and promoted osteogenic differentiation in combination with upregulation of osteogenic genes. The mRNA expression levels of key genes that are closely associated with osteogenesis were verified by qPCR (Fig. S6). For cell components, the DEGs were enriched in early endosomes and adherence junctions (Fig. 3e). For molecular functions (MFs), DEGs were significantly enriched in transmembrane receptor protein tyrosine kinase activity and Smad protein binding, among which growth factor activity, structural extracellular matrix constituents, and collagen binding were the most likely to play significant roles in osteogenesis (Fig. 3d). Furthermore, a low concentration of ZA most likely promoted osteogenesis via the PI3K-Art, JAK-STAT, and Wnt signaling pathways, according to Kyoto Encyclopedia of Genes and Genomes (KEGG)-enriched gene network analysis (Fig. 3f).

### Surgical procedure

**Fig. 4:**
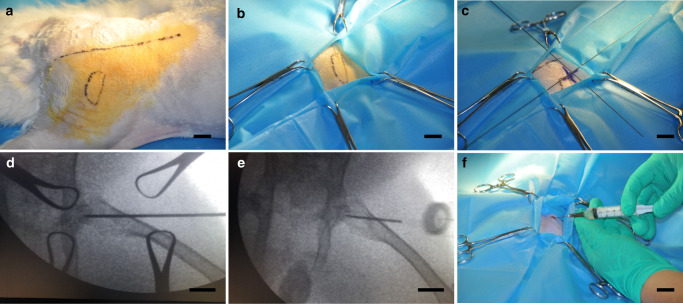
Surgical procedure. **a** Skin preparation and disinfection after anesthetization. **b** Sterile sheet preparation at the surgical site. **c** Femoral head orientation using a homemade surgical guide. **d** Core decompression using a needle. **e** Injection orientation. **f** Injection of ZA-loaded GPCL through an 18G needle. Scale bar: 1 cm

Materials loaded with zoledronic acid were evaluated in animal models of CD with osteonecrotic femoral heads. Due to the injectability of this polymer, the entire surgical procedure was conducted percutaneously, allowing the greatest protection of soft tissue and superior postoperative wound care. Furthermore, this type of surgery decreased the possibility of infection because it is minimally invasive. After CD, approximately 0.2 mL of ZA-loaded GPCL was injected through an 18G needle (Video S2). The injection site was again sterilized, after which the lesions were covered with an antiseptic dressing (Fig. 4a–f). The contralateral side was treated in the same manner but without the injection of material. After being resuscitated from anesthesia, the rabbits were housed in separate cages and fed a standard diet. After 2 months, all experimental animals were sacrificed, and bilateral femoral head samples were collected. No animal deaths or serious adverse reactions were observed within the 2-month observation period.

### Therapeutic effects in vivo

**Fig. 5:**
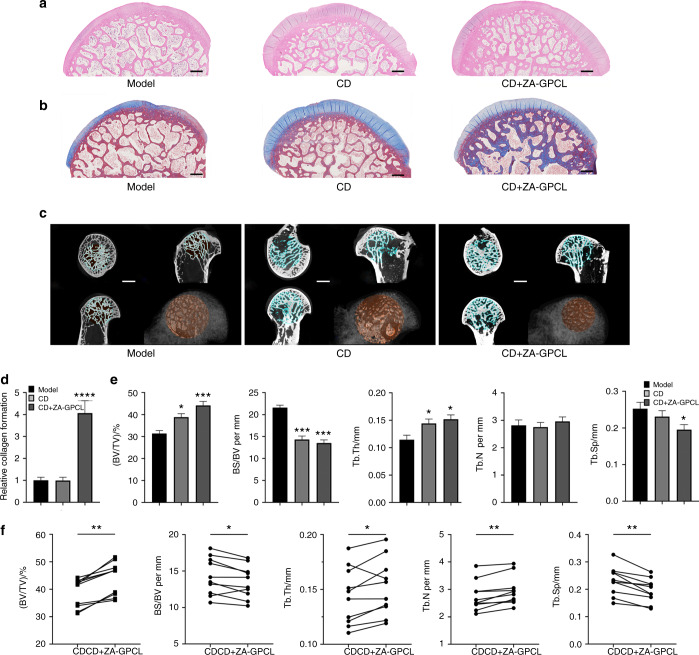
General therapeutic effect in vivo. **a** HE-stained decalcified femoral head sections. Scale bars: 500 μm. **b** Masson’s staining of decalcified femoral head sections. Scale bars: 500 μm. **c** Sectional and 3D-reconstructed micro-CT images demonstrating the therapeutic effects on rabbit femoral heads. Scale bars: 1 mm. **d** Quantification of collagen formation in Masson’s-stained sections. **e** Quantification of BV/TV, BS/BV, Tb. N, Tb.Th, and Tb.Sp values of the femoral heads in the different treatment groups. **f** Paired analysis of BV/TV, BS/BV, Tb. N, Tb.Th, and Tb.Sp of the bilateral femoral heads of rabbits. All values represent the mean ± SEM (*n* ≥ 6)

In the present study, an injectable and biodegradable drug delivery strategy was used to treat GA-ONFH. Decalcified HE sections and micro-CT images of the femoral head in the simple CD and CD+ZA-GPCL groups demonstrated differences in appearance compared with that in the model group. Trabecular bone in the model group, including that in the subchondral region, was considerably thinner and weaker than the bone in the other groups, indicating that those animals suffered more severe bone loss (Fig. 5a). Masson’s trichrome-stained sections exhibited deeper and larger blue staining in the CD+ZA-GPCL group than in the model or CD groups, indicating that more collagen (COL) had formed in the CD+ZA-GPCL group (Fig. 5b). Quantification of the sections demonstrated that femoral heads treated with CD+ZA-GPCL exhibited approximately threefold more collagen formation than those in the model group or simple CD group (*P* = 0.001), which was important in strengthening the mechanical properties of trabecular bone (Fig. 5d). Furthermore, the bone trabeculae in the CD and CD+ZA-GPCL groups were arranged more tightly and orderly than the bone trabeculae in the model group. Consistent with the pathological sections, 3D and sectional micro-CT images revealed that the trabecular separation of femoral heads in the model group was significantly larger than that in the CD and CD+ZA-GPCL groups (Fig. 5c). Further quantification revealed that in the simple CD and CD+ZA-GPCL groups, the BV/TV, BS/BV, Tb.Th, and Tb.Sp values changed to a smaller extent than in the other groups. The BV/TV in the CD (38.77% ± 1.642%) and CD+ZA-GPCL groups (44.09% ± 1.895%) was significantly higher than that in the model group (31.32% ± 1.403%) (*P*_CD vs model_ = 0.021, *P*_CD+ZA-GPCL vs model_ = 0.001). The BS/BV in the CD (14.30 ± 0.806 4/mm) and CD+ZA-GPCL (13.52 ± 0.707 3/mm) groups was significantly lower than that in the model group (21.58 ± 0.572 5/mm) (*P*_CD vs model_ = 0.005, *P*_CD+ZA-GPCL vs model_ = 0.005). Tb.Th in the CD group (0.144 0 ± 0.008 226 mm) and CD+ZA-GPCL (0.151 8 ± 0.008 148 mm) groups was significantly higher than that in the model group (0.114 5 ± 0.008 376 mm) (*P*_CD vs model_ = 0.041, *P*_CD+ZA-GPCL vs model_ = 0.016). Tb.Sp in the CD (0.230 8 ± 0.016 47 mm) and CD+ZA-GPCL (0.195 2 ± 0.013 82 mm) groups was significantly lower than that in the model group (0.252 4 ± 0.017 80 mm) (*P*_CD vs model_ = 0.241, *P*_CD+ZA-GPCL vs model_ = 0.021). Tb.N in the CD (2.753 ± 0.171 1/mm) and CD+ZA-GPCL (2.957 ± 0.166 5/mm) groups was not significantly different from that in the model group (2.815 ± 0.198 6/mm) (*P*_CD vs model_ = 0.859, *P*_CD+ZA-GPCL vs model_ = 0.374) (Fig. 5e). Because simple CD and CD+ZA-GPCL operations were performed bilaterally on the same experimental animal, paired tests were conducted. CD+ZA-GPCL exhibited an overall advantage over CD in BV/TV (*P*  = 0.005), BS/BV (*P* = 0.022), Tb.Th (*P* = 0.037), Tb.N (*P* = 0.005) and Tb.Sp (*P* = 0.005) (Fig. 5f). These data suggested that CD could reverse bone loss to decrease the possibility of femoral head collapse. Furthermore, the trabecular bone that was treated with CD+ZA-loaded GPCL exhibited a thicker and denser arrangement with 3-fold greater collagen formation relative to that in the other groups, suggesting that CD+ZA-GPCL is effective in inhibiting bone resorption and increasing the mechanical properties of the femoral head. Thus, this strategy reduced the possibility of femoral head collapse by forming more solid support for subchondral bone and cartilage than other materials.

**Fig. 6:**
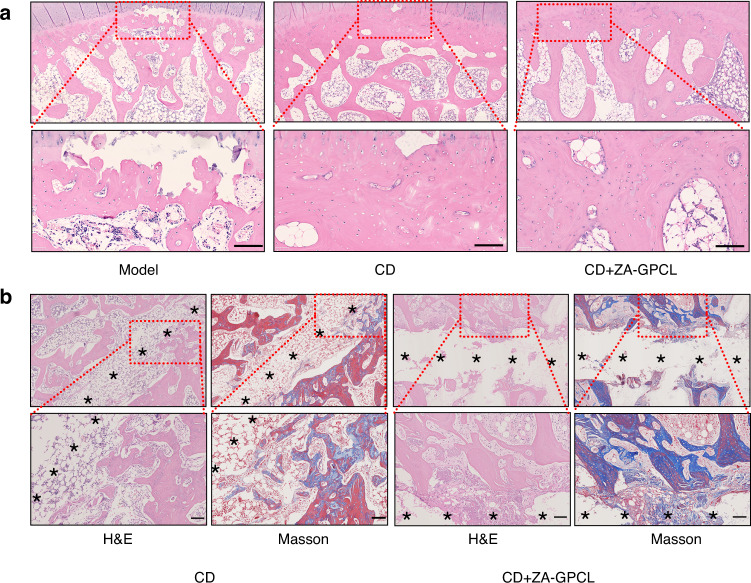
Local therapeutic effect in vivo. **a** HE-stained subchondral bone. **b** HE- and Masson’s-stained bone regeneration in the core decompression tunnel. Black stars indicate CD tunnels. Undegraded GPCL was dissolved during the section staining process. Scale bars: 100 μm

Within the subchondral region, the bone trabeculae in the model group were more fragile, and mechanical support for this cartilage was weakened or even completely lost. The subchondral bone in the CD and CD+ZA-GPCL groups was regular in not only thickness but also histological appearance. In addition, HE-stained sections demonstrated that the number of empty lacunae was decreased in the CD+ZA-GPCL group compared with the other two groups (Fig. 6a). The empty CD tract was full of bone marrow in the CD group, while ZA-GPCL filled the tract in the CD+ZA-GPCL group. Femoral heads that were treated with glucocorticoids exhibited low regeneration due to damage to MSCs and other osteoprogenitors. However, compared with simple CD treatment, enhanced osteogenicity and increased collagen formation around the tract suggested that ZA-GPCL could accelerate bone regeneration to augment CD and prevent femoral head collapse (Fig. 6b).

## Discussion

ONFH is not a simple disease caused by simple cell dysfunction or gene misexpression. It seems that the local imbalance in bone homeostasis is caused by the overuse of glucocorticoids. This imbalance involves both bone cells and the microenvironment. Most importantly, the logical relationship between microenvironment changes and bone cell dysfunction should be clarified to uncover the pathogenesis of ONFH. Before that, symptomatic treatments were important in helping local homeostasis reconstruction.

CD is a strategy that aims to activate injury-repair reactions by destroying the sclerotic belt. It is thought that CD could enhance blood microcirculation and promote vascularization of the necrotic region in the femoral head.^44^ The procedure is usually performed with core drills targeting the necrotic zone. After drilling, necrotic bone is usually removed using a curette. On the one hand, this procedure activates injury-repair reactions in the femoral head. On the other hand, the removal of necrotic bone bypasses necrotic bone resorption and ameliorates bone regeneration. A series of animal studies and clinical studies have established the effectiveness of this method in treating early-stage ONFH.^45–47^ However, this method also has some shortcomings. With widely varying rates of success, the technique is associated with various complications, including an increased risk of collapse and subtrochanteric fractures requiring a surgical incision.^48^ The combination of therapeutic agents and biomaterials inspired improvements for CD.^49,50^ Various adjunctive therapies have been investigated in combination with CD to ameliorate therapeutic outcomes, such as stem cell-seeded scaffolds^17^ and vascularized bone grafts.^51^ These strategies require advanced fabrication, which is inconvenient and is associated with practical difficulties. In addition, vascularized bone grafts can result in secondary injury to patients, and the use of stem cells may raise ethical concerns. Therefore, a cell-free therapy that can be used easily is required for CD.

There remains controversy regarding the use of BP in ONFH treatment.^52^ In clinical trials, Lee et al. concluded that a single infusion of zoledronate did not prevent the collapse of the femoral head or reduce the need for THA.^53^ Friedl et al. showed that zoledronic acid in combination with implants might have promising outcomes for ONFH treatment.^54^ Different clinical outcomes might be related to the drug administrative method because the decreased blood supply in the osteonecrotic region is a barrier for achieving effective drug concentrations. The ZA-loaded GPCL in combination with CD was designed for early-stage ONFH to solve these problems. Injectable GPCL containing ZA exhibited superior characteristics for this type of clinical application. ZA, a high-performance osteoclast inhibitor, can reduce the number of overactive osteoclasts and thereby regulate bone resorption. In addition, it has been established that low concentrations of ZA can promote osteogenesis via different mechanisms. In this study, after one week of 10^−8^ mol·L^−1^ ZA administration, hMSCs showed osteogenetic superiority over those in the control group (Fig. S5a, c). ALP staining and RT-qPCR analysis of BLGAP (OCN) and RUNX2 on the 7th day confirmed the hypothesis that ZA played a crucial osteogenic role at an early stage. In addition, Alizarin Red S staining on the 14th day revealed that ZA significantly promoted calcium nodule formation (Fig. S5b). Accordingly, the RT-qPCR results indicated the importance of properly prolonging the administrative time of ZA (Fig. S5d). Therefore, the combination of ZA and GPCL might be a solution to maximize the benefits of ZA, not only to inhibit bone resorption but also to promote osteogenesis. Regarding the detailed osteogenetic mechanism of ZA, the RNA-seq results indicated that ZA inhibited certain negative osteogenic genes, including OSTN, RANBP3L, AXIN2, and MMRN1, while upregulating positive osteogenic genes, including ALDH3A1 and NRARP, at the transcriptional level (Fig. S6). From a chemical perspective, ZA has a strong affinity for calcium ions, which might be important for bone formation. The promotion of calcium nodule formation and upregulation of BLGAP expression provided convincing evidence for this hypothesis (Fig. S5b–d). When exposed on the surface of GPCL in vivo, ZA could act as an anchor to accelerate calcium deposition before it is released from the material, which might contribute to the increased osteogenesis observed near the CD track in this study. In addition, there is another potential mechanism by which ZA promotes bone formation by regulating angiogenesis, according to some in vivo studies.^55^ Moreover, ZA could be a routine additive to orthopedic biomaterials to regulate implant-induced osteoclast proliferation.

GPCL melted at a relatively low temperature of approximately 50 °C and solidified at a moderate rate, suggesting its capability of being loaded with high temperature-sensitive agents. Furthermore, the moderate rate of solidification enables surgeons to conduct injections at a more leisurely pace. Compared to hydrogels,^56^ microspheres,^57,58^ or soft scaffolds,^59^ GPCL also exhibited excellent mechanical properties and acted as hard tissue. The mechanical properties of GPCL were found to be similar to those of cancellous bone, and GPCL is capable of acting as a scaffold,^60^ providing mechanical support in the tunnel. This characteristic prevents microfractures around the tunnel. In addition, its biodegradability plays a role in avoiding implant removal and may contribute to its sustained release capability, which is superior to that of metal implants.^61^ In combination with ZA-GPCL, CD not only inhibited bone loss but also increased collagen expression in the cancellous bone of the femoral head, decreasing the possibility of collapse by strengthening cancellous bones. In general, the use of CD in combination with an injection of ZA-loaded GPCL exhibited satisfactory outcomes for the treatment of early-stage ONFH, indicating a potential solution for the prevention of femoral head collapse.

Although the use of ZA-loaded GPCL in combination with CD exhibited promising therapeutic properties for the treatment of early-stage ONFH, many problems remain to be resolved. Zoledronic acid has been reported to promote osteogenesis and increase bone strength both in vivo and in vitro.^62–66^ However, the dose and frequency of ZA administration require additional exploration. The RNA sequencing results revealed that the osteogenic effect of ZA and its mechanism of action were unclear during the first 7 days of intervention. Unlike the sequencing results from day 14, it was difficult to identify osteogenic-related molecules or signaling pathways, although 73 genes were differentially expressed (Fig. S7). By comparing the results at different time points, we hypothesize that the osteogenic effect of ZA on hMSCs may be closely related to the duration of the intervention. A relatively longer administrative duration may answer this question. However, the in vitro release and degradation assay results may readily be influenced by mechanical impact. Long-term in vivo release and degradation in femoral head tissues should be explored further before clinical application. Various methods can be used to establish GA-ONFH animal models. Considering the immunosuppressive and metabolic effects of glucocorticoids, animals such as rabbits or rats are likely to die, and approximately one-quarter of the glucocorticoid-treated rabbits in the present study were sacrificed before CD surgery. In addition, the hip joint biomechanics of quadrupeds such as rats, mice, and rabbits are quite different from those of humans. Unlike quadrupeds, humans walk, stand, and run in an upright orientation. Such biomechanical characteristics result in humans suffering a greater incidence of ONFH, with more microfractures and collapse compared with quadruped mammals. To date, there have not been any ONFH animal models to mimic the collapse that occurs in humans, which makes collapse prevention research difficult. Furthermore, scoring systems to evaluate feel and function, such as pain or Harris scores, cannot be used in existing animal models, which is a critical limitation in related research. Consequently, it is important to establish a GA-ONFH animal model that shares similar biomechanical characteristics with humans for further ONFH animal studies. Considering the mechanical influence on femoral head collapse, glucocorticoid induction combined with pad implantation beneath the inner acetabulum in quadrupeds may be effective for imitating the natural course of GA-ONFH in humans, which deserves more in-depth evaluation.

## Conclusion

In summary, an injectable GPCL polymer can be loaded with thermolabile therapeutic agents and serve as a scaffold that provides mechanical support. Combined with CD, injection of the ZA-GPCL polymer induces a positive therapeutic effect on early-stage ONFH.

## Materials and methods

### Materials

All chemicals were used as purchased without further purification. ε-Caprolactone, glycerol, PEG (Mw ~800), and dichloromethane were purchased from Macklin (Shanghai, China). Tin 2-ethyl hexanoate was obtained from Aladdin (Shanghai, China). Diethyl ether was obtained from Chron Chemicals (Chengdu, China). Methanol was obtained from Hengxing Chemical Reagents (Tianjin, China). Alpha-minimum essential medium (α-MEM) and fetal bovine serum (FBS) were acquired from Gibco (Grand Island, USA). Penicillin–streptomycin was obtained from HyClone (Utah, USA). MC3T3-E1 and human bone marrow mesenchymal stem cells were bought from Procell Life Science & Technology (Wuhan, China). Dexamethasone, vitamin C, and β-glycerophosphate disodium salt pentahydrate were purchased from Solarbio (Beijing, China). Zoledronic acid was purchased from Novartis (Basel, Switzerland), TRIzol reagent was purchased from Invitrogen (Carlsbad, USA), and vancomycin was purchased from Eli Lilly and Company (Indianapolis, Indiana). Vancomycin ELISA kits were purchased from Jianglai Biotech (Shanghai, China). Lipopolysaccharide (LPS) was obtained from Sigma Aldrich (St. Louis, MO, USA), and methylprednisolone (MPS) was purchased from Pfizer (New York, USA). New Zealand white rabbits were purchased from Keao Biotech (Xi'an, China). Pentobarbital sodium was obtained from Aikonchem (Nanjing, China), and PBS-EDTA (0.5 mol·L^−^^1^ EDTA, pH 8.0) and paraformaldehyde were obtained from Aladdin (Shanghai, China). Kirschner wires for core decompression surgery were bought from Gemmed (Suzhou, China). Primers for qPCR were designed and synthesized by Sangon Biotech (Shanghai, China). Universal SYBR Green Fast qPCR Mix was acquired from ABclonal (Wuhan, China). StarScript II RT mix, RT Reaction Mix, and DEPC-ddH_2_O were purchased from GenStar (Beijing, China). Alkaline phosphatase staining kits were purchased from WAKO (Kyoto, Japan), while the Alizarin Red S staining solution was purchased from Beyotime (Shanghai, China).

### Polymer synthesis

To modify PCL (GPCL), 11.4 g of ε-caprolactone, 0.3 g of glycerin, and 0.1 g of stannous octoate were mixed using a planetary mixer (Thinky ARE-300) at 2 000  r·min^−1^ for 2 min. The solution was then heated in a vacuum oven at 120 °C for 24 h. After the temperature had fallen to ~80 °C, 0.59 g of dichloromethane was added to the liquid and mixed thoroughly. This liquid was poured slowly into ice-cold diethyl ether (Et_2_O) to condense into a solid. The resultant solid was then heated to 60 °C to form a liquid. The liquid was then precipitated in ice-cold methanol (MeOH). The Et_2_O-MeOH washing process was repeated twice to obtain the final product. For unmodified PCL, no glycerin was added. For GPCL/PEG, PEG was directly mixed with the product at 60 °C at a weight ratio of 1:4. Tests were repeated at least three times.

### Properties of the polymer

Compressive test specimens were fabricated into cylinders (30 mm long, 19.85 mm diameter) by casting in an aluminum mold. Testing was performed using an AGS-X at a rate of 10 mm per min. Specimens were stored at 37 °C. DSC curves were prepared using a TA DSC Q2000 at a rate of 3 °C per min. Viscosity was measured using an NDJ-9S rotational viscometer in 10 mL glass sample bottles at a speed of 30 r·min^−1^. Thermal images were recorded using a FLIR A6703sc 470. Macroscopic images of the polymers were recorded using a Canon EOS 5D digital camera. ^1^H-NMR spectra were obtained using an Avance III HD 600 MHz.

### In vitro cell viability

Extracts of GPCL and GPCL/PEG were created according to the requirements of ISO 10993-12. GPCL and GPCL/PEG polymers were sterilized using an autoclave (103.4 kPa, 121.3 °C, 30 min) and shaped into 5 × 25 × 20 mm^3^ cuboids. Each sample was individually immersed in 5 mL of complete α-MEM supplemented with 9% FBS and 1% penicillin–streptomycin at 37 °C for 72 h. MC3T3-E1 cells were seeded in 96-well plates at a density of ~3 × 10^3^ cells per well. After 8 h of culture, the medium was removed, which represented a polymer extract. Complete culture medium without extract was used as a control. Images of each well were acquired every 6 h using a live-cell imaging system (Cytation5, BioTek, USA). Each experiment was repeated five times.

### Drug release assay

One microgram of vancomycin was mixed into the GPCL polymer before the material was formed into cylindrical implants (15 mm long, 5 mm diameter), and then immersed in 10 mL of PBS (pH 7.4) at 37 °C. A 200 μL aliquot was collected to measure the drug concentration after 1, 7, 14, and 21 days. The same volume of PBS was added to the system after the collection of each sample. Concentrations of vancomycin at different time points were measured using an ELISA kit according to the manufacturer’s instructions. Each analysis was repeated five times.

### Effect of low-concentration ZA on hMSCs in vitro

P4 hMSCs were seeded in 6-well plates at a density of approximately 10^4^ cells per well. After 24 h, the cells (approximately 70% confluence) were divided into two groups, and each group received different interventions. OIM was composed of α-MEM supplemented with 10% FBS and 1% penicillin–streptomycin and contained 10 nmol·L^−1^ dexamethasone, 50 mg·L^−1^ vitamin C, and 5 mmol·L^−1^ β-glycerophosphate. Cells in the control group were cultured with OIM only. The ZA group was treated with OIM plus 10^−8^ mol·L^−1^ ZA. The culture medium was refreshed every 2 days, and the cells were washed three times with PBS each time. Cells in the different groups were harvested and stored in TRIzol reagent for RNA sequencing after 7 and 14 days.

### RNA-seq and differentially expressed gene analysis

The DNBSEQ platform was used for sequencing, and the results produced 150 bp (PE150) paired-end reads. Preprocessing of the raw sequencing data was performed, and low-quality and short reads (≤20 bp) were removed. Following quality control, clean reads were aligned to the reference genome based on HISAT. The R package DESeq2 was used to perform differential gene expression analysis. In the present study, DEGs were defined as those with *P* < 0.05 and |log_2_fold change|≥1.

### GO function and KEGG pathway enrichment analysis

GO and KEGG enrichment analyses were conducted using the cluster Profiler R package. Items from biological process, molecular function, cellular component, and KEGG pathways for which *P* < 0.05 were considered significantly enriched.

### Real-time quantitative PCR

After RNA sequencing, 1 μg of total RNA was reverse transcribed into cDNA using 1 μL of StarScript II RT mix, 10 μL of 2× RT Reaction Mix (with primer), and DEPC-ddH_2_O. Reverse transcription was performed at 42 °C for 15 min and then finally terminated at 85 °C after 5 min. qPCR was then performed in triplicate with 1 μL of cDNA, 10 μL of 2× Universal SYBR Green Fast qPCR Mix, 0.4 μL of 10 μmol·L^−1^ forward primer, 0.4 μL of 10 μmol·L^−1^ reverse primer, and 8.2 μL of DEPC-ddH_2_O according to the manufacturer’s instructions using a QuantStudio^®^ 5 Real-Time PCR System (Thermo Fisher Scientific, MA, USA). qPCR was conducted as follows: 3 min at 95 °C, followed by 42 cycles of 5 s at 95 °C and 30 s at 60 °C. Cycle threshold values were recorded for target genes, after which target mRNA levels were normalized to the level of glyceraldehyde 3-phosphate dehydrogenase mRNA. Relative gene expression was calculated using the 2^−Δ^^Δ^^Ct^ method. The primer sequences used in this assay are shown in Table S1.

### Cell culture and staining

P4 hMSCs were seeded in 6-well plates at a density of approximately 10^4^ cells per well. Classification and treatment methods are described above. On day 7, ALP staining kits were used according to the manufacturer’s instructions. On day 14, Alizarin Red S staining was performed using 0.2%, (pH 8.3) Alizarin Red S staining solution. Microphotographs were taken with an inverted light microscope (Olympus Corporation, Tokyo, Japan).

### ONFH animal model

Female New Zealand white rabbits weighing 4.0–5.0 kg were injected intravenously with LPS at a dose of 10 μg·kg^−1^ body weight via the auricular vein. After 24 h, three intramuscular injections of MPS were administered at a dose of 20 mg·kg^−1^ body weight every 24 h.^67^ Each rabbit was housed in a separate cage and fed a standard diet for 8 weeks.

### Animal surgery

Ten rabbits with confirmed ONFH were used for the surgery, while the rest were used in the model group and further housed. Surgery was performed 8 weeks after the administration of LPS. Before the surgery, 1.0 mg of ZA was added at approximately 60 °C to 1 mL of liquid GPCL and mixed using two injectors to ensure that the ZA was uniformly distributed within the polymer. Rabbits were anesthetized with pentobarbital sodium at a dose of 0.03 g·kg^−1^ body weight. After the skin was prepared, the animals were placed in a prone position. The surgical area was disinfected three times with iodophor and surgical drapes were placed around the site of implantation. The femoral head was located with the guidance of X-ray imaging. CD was performed on the greater trochanter, ending percutaneously at the subchondral zone, with the track going from the greater trochanter into the subchondral bone beneath the cartilage through the femoral neck. The process was completed using 1.0 mm Kirschner wires with the help of both anteroposterior and frog-leg lateral positioning correction. For each animal, the left femoral head received simple CD, while the right femoral head received CD and an injection of approximately 0.2 mL of ZA-loaded polymer. Injections were administered using a syringe with an 18G needle after ZA-GPCL was heated to a liquid. The dissolved ZA-GPCL filled the CD tunnel and part of the marrow cavity. After surgery, bupivacaine and penicillin G were administered for postoperative analgesia and resistance to infection. All rabbits were sacrificed 2 months after CD surgery, and the femoral heads were collected and stored in 4% paraformaldehyde for additional micro-CT scanning and histological slicing. The experimental protocol was approved by the Animal Experiment Ethics Committee of Xi’an Jiaotong University, China (Approval No. XJTULAC2019-1287).

### Micro-CT analysis

Femoral head samples were scanned using a Y. Cheetah micro-CT (Yxlon, Hamburg, Germany) at a voltage of 80 kV and a current of 62.5 mA. Scanning data were reconstructed and analyzed using VGStudio MAX 3.0 (Volume Graphics, Heidelberg, Germany).

### Histological evaluation

Femoral head samples were decalcified using PBS-EDTA (0.5 mol·L^−1^ EDTA, pH 8.0) at room temperature after micro-CT scanning was performed. The decalcifying fluid was changed every other day until the samples were suitable for slicing. H&E and Masson’s trichrome staining were performed on 4-μm-thick paraffin sections, which were and then scanned using a Nano Zoomer (Hamamatsu Photonics, Shizuoka, Japan). Sections of skin-muscle samples were H&E-stained. The slides were viewed with NDP view 2 software (Hamamatsu Photonics, Shizuoka, Japan). All histological sections were analyzed using ImageJ software (NIH, Bethesda, USA). The evaluation was conducted by a pathologist who was blinded to the group assignments.

### Statistical analysis

The data were statistically analyzed, including a two-tailed *t* test or Mann–Whitney test whenever applicable for independent samples. To analyze the micro-CT results, a paired two-tailed *t* test or Wilcoxon test was performed whenever applicable for paired samples. In vitro cell biocompatibility, qPCR, and collagen expression results were analyzed using an independent two-tailed *t* test. The fibrous layer thickness was analyzed using a Kruskal–Wallis test. All statistical analyses were conducted using IBM SPSS Statistics 24 software (IBM, New York, USA). All results are presented as the mean ± SEM. *P < 0.05, **P < 0.01, and ***P < 0.001 were considered significant.

## Supplementary information

Supplementary information

Certification

Animal care

Video S1

Video S2

## Data Availability

The datasets generated and/or analyzed during the current study are available from the corresponding author upon reasonable request.

## References

[1] Israelite C (2005). Bilateral core decompression for osteonecrosis of the femoral head. Clin. Orthop. Relat. Res..

[2] Yoon BH (2019). Etiologic classification criteria of ARCO on femoral head osteonecrosis part 1: glucocorticoid-associated osteonecrosis. J. Arthroplast..

[3] Hungerford DS (2002). Osteonecrosis: avoiding total hip arthroplasty. J. Arthroplast..

[4] Mont MA, Salem HS, Piuzzi NS, Goodman SB, Jones LC (2020). Nontraumatic osteonecrosis of the femoral head: where do we stand today?: a 5-year update. J. Bone Joint Surg. Am..

[5] Zhao D (2020). Guidelines for clinical diagnosis and treatment of osteonecrosis of the femoral head in adults (2019 version). J. Orthop. Transl..

[6] Zhang Q (2021). Surgical procedures for hip joint preservation for osteonecrosis of the femoral head: a bibliometric analysis. BioMed. Res. Int..

[7] Uesugi Y (2018). Quality of life of patients with osteonecrosis of the femoral head: a multicentre study. Int. Orthop..

[8] Lavernia CJ, Sierra RJ, Grieco FR (1999). Osteonecrosis of the femoral head. J. Am. Acad. Orthop. Surg..

[9] Molloy IB, Martin BI, Moschetti WE, Jevsevar DS (2017). Effects of the length of stay on the cost of total knee and total hip arthroplasty from 2002 to 2013. J. Bone Joint Surg. Am..

[10] Pollock, M., Somerville, L., Firth, A. & Lanting, B. Outpatient total hip arthroplasty, total knee arthroplasty, and unicompartmental knee arthroplasty: a systematic review of the literature. *JBJS Rev.* **4**, e4 (2016). 10.2106/JBJS.RVW.16.00002 28060788

[11] Swarup I (2018). Implant survival and patient-reported outcomes after total hip arthroplasty in young patients. J. Arthroplast..

[12] Cohen-Rosenblum A, Cui Q (2019). Osteonecrosis of the femoral head. Orthop. Clin. North Am..

[13] Yu X (2018). Effectiveness of various hip preservation treatments for non-traumatic osteonecrosis of the femoral head: a network meta-analysis of randomized controlled trials. J. Orthop. Sci..

[14] Goodman SB (2018). The biological basis for concentrated iliac crest aspirate to enhance core decompression in the treatment of osteonecrosis. Int Orthop..

[15] Andronic O, Weiss O, Shoman H, Kriechling P, Khanduja V (2021). What are the outcomes of core decompression without augmentation in patients with nontraumatic osteonecrosis of the femoral head?. Int. Orthop..

[16] Marker DR, Seyler TM, Ulrich SD, Srivastava S, Mont MA (2008). Do modern techniques improve core decompression outcomes for hip osteonecrosis?. Clin. Orthop. Relat. Res..

[17] Maruyama M (2018). The effects of a functionally-graded scaffold and bone marrow-derived mononuclear cells on steroid-induced femoral head osteonecrosis. Biomaterials.

[18] Zhang CQ (2011). Free vascularised fibular graft for post-traumatic osteonecrosis of the femoral head in teenage patients. J. Bone Joint Surg. Br..

[19] Veillette CJ, Mehdian H, Schemitsch EH, McKee MD (2006). Survivorship analysis and radiographic outcome following tantalum rod insertion for osteonecrosis of the femoral head. J. Bone Joint Surg. Am..

[20] Hamada H, Takao M, Sakai T, Sugano N (2018). Subchondral fracture begins from the bone resorption area in osteonecrosis of the femoral head: a micro-computerised tomography study. Int. Orthop..

[21] Kawano K (2020). Differences in the microarchitectural features of the lateral collapsed lesion between osteonecrosis and subchondral insufficiency fracture of the femoral head. Bone.

[22] Baba S (2020). Quantitative evaluation of bone-resorptive lesion volume in osteonecrosis of the femoral head using micro-computed tomography. Joint Bone Spine.

[23] Weinstein RS (2011). Glucocorticoid-induced bone disease. N. Engl. J. Med..

[24] Mhaskar R, Djulbegovic B (2018). Bisphosphonates for patients diagnosed with multiple myeloma. JAMA.

[25] Singer FR (2009). Paget disease: when to treat and when not to treat. Nat. Rev. Rheumatol..

[26] Cremers S, Drake MT, Ebetino FH, Bilezikian JP, Russell RGG (2019). Pharmacology of bisphosphonates. Br. J. Clin. Pharm..

[27] Reid IR (2015). Short-term and long-term effects of osteoporosis therapies. Nat. Rev. Endocrinol..

[28] Morgan GJ (2010). First-line treatment with zoledronic acid as compared with clodronic acid in multiple myeloma (MRC Myeloma IX): a randomised controlled trial. Lancet.

[29] Raje N (2018). Denosumab versus zoledronic acid in bone disease treatment of newly diagnosed multiple myeloma: an international, double-blind, double-dummy, randomised, controlled, phase 3 study. Lancet Oncol..

[30] Maruyama M (2021). The efficacy of lapine preconditioned or genetically modified IL4 over-expressing bone marrow-derived mesenchymal stromal cells in corticosteroid-associated osteonecrosis of the femoral head in rabbits. Biomaterials.

[31] Yue S, He H, Li B, Hou T (2020). Hydrogel as a biomaterial for bone tissue engineering: a review. Nanomaterials (Basel).

[32] Hasani-Sadrabadi MM (2020). An engineered cell-laden adhesive hydrogel promotes craniofacial bone tissue regeneration in rats. Sci. Transl. Med..

[33] Benmassaoud MM, Gultian KA, DiCerbo M, Vega SL (2020). Hydrogel screening approaches for bone and cartilage tissue regeneration. Ann. N. Y. Acad. Sci..

[34] Kondiah PJ (2016). A review of injectable polymeric hydrogel systems for application in bone tissue engineering. Molecules.

[35] He M (2020). Porous tantalum rod implantation is associated with low survival rates in patients with type C2 osteonecrosis of the femoral head but has no effect on the clinical outcome of conversion total hip arthroplasty: a retrospective study with an average 8-year follow-up. BMC Musculoskelet. Disord..

[36] Mondal D, Dixon SJ, Mequanint K, Rizkalla AS (2017). Mechanically-competent and cytocompatible polycaprolactone-borophosphosilicate hybrid biomaterials. J. Mech. Behav. Biomed. Mater..

[37] Ciardelli G (2005). Blends of poly-(epsilon-caprolactone) and polysaccharides in tissue engineering applications. Biomacromolecules.

[38] Camarero-Espinosa S, Moroni L (2021). Janus 3D printed dynamic scaffolds for nanovibration-driven bone regeneration. Nat. Commun..

[39] Pirosa A (2021). An in vitro chondro-osteo-vascular triphasic model of the osteochondral complex. Biomaterials.

[40] Porrelli D (2021). Antibacterial electrospun polycaprolactone membranes coated with polysaccharides and silver nanoparticles for guided bone and tissue regeneration. ACS Appl. Mater. Interfaces.

[41] Sosnoski DM, Gay CV (2007). Evaluation of bone-derived and marrow-derived vascular endothelial cells by microarray analysis. J. Cell Biochem..

[42] Thomas G (2003). Osteocrin, a novel bone-specific secreted protein that modulates the osteoblast phenotype. J. Biol. Chem..

[43] Zhou Y (2019). The methylation of Notch1 promoter mediates the osteogenesis differentiation in human aortic valve interstitial cells through Wnt/beta-catenin signaling. J. Cell Physiol..

[44] Wang BL (2010). Treatment of nontraumatic osteonecrosis of the femoral head with the implantation of core decompression and concentrated autologous bone marrow containing mononuclear cells. Arch. Orthop. Trauma Surg..

[45] Maruyama M (2020). The efficacy of core decompression for steroid-associated osteonecrosis of the femoral head in rabbits. J. Orthop. Res..

[46] Yang P (2014). Core decompression in combination with nano-hydroxyapatite/polyamide 66 rod for the treatment of osteonecrosis of the femoral head. Arch. Orthop. Trauma Surg..

[47] Mont MA, Cherian JJ, Sierra RJ, Jones LC, Lieberman JR (2015). Nontraumatic osteonecrosis of the femoral head: where do we stand today? A ten-year update. J. Bone Joint Surg. Am..

[48] Mont, M. A., Ragland, P. S. & Etienne, G. Core decompression of the femoral head for osteonecrosis using percutaneous multiple small-diameter drilling. *Clin. Orthop. Relat. Res*.131–138 (2004). 10.1097/01.blo.0000150128.57777.8e 15577477

[49] Kong L (2020). Repair materials for bone defects of the tibial plateau in primary total knee arthroplasty. Mater. Express.

[50] Li X (2020). Combined study on the action and mechanism of G-Rg1/Sr-CaS bone substitute material for ossification and pro-vascularization. Mater. Express.

[51] Zhao D (2016). Vascularized bone grafting fixed by biodegradable magnesium screw for treating osteonecrosis of the femoral head. Biomaterials.

[52] Li D, Yang Z, Wei Z, Kang P (2018). Efficacy of bisphosphonates in the treatment of femoral head osteonecrosis: a PRISMA-compliant meta-analysis of animal studies and clinical trials. Sci. Rep..

[53] Lee YK (2015). Does zoledronate prevent femoral head collapse from osteonecrosis? A prospective, randomized, open-label, multicenter study. J. Bone Joint Surg. Am..

[54] Friedl G (2009). The effect of a single infusion of zoledronic acid on early implant migration in total hip arthroplasty. A randomized, double-blind, controlled trial. J. Bone Joint Surg. Am..

[55] Ramasamy SK (2016). Blood flow controls bone vascular function and osteogenesis. Nat. Commun..

[56] Wu Y (2020). Role of hydrogels in bone tissue engineering: how properties shape regeneration. J. Biomed. Nanotechnol..

[57] Zhang X (2020). Hyperbranched polymer micelles with triple-stimuli backbone-breakable iminoboronate ester linkages. Chin. Chem. Lett..

[58] Yang W (2020). Fibrin scaffolds embedded with sonic hedgehog/chitosan microspheres for recovery of spinal cord injury in rats. Mater. Express.

[59] Huang K, Liu G, Gu Z, Wu J (2020). Tofu as excellent scaffolds for potential bone regeneration. Chin. Chem. Lett..

[60] Zhao D, Ma Z (2020). Application of biomaterials for the repair and treatment of osteonecrosis of the femoral head. Regen. Biomater..

[61] Lu X (2020). Nanoparticle shaped titanium promotes osteogenic differentiation of bone mesenchymal stem cells through integrin/integrin linked kinase/glycogen synthase kinase-3β axis. J. Biomed. Nanotechnol..

[62] Little DG (2003). Zoledronic acid prevents osteopenia and increases bone strength in a rabbit model of distraction osteogenesis. J. Bone Min. Res..

[63] Zwolak P, Farei-Campagna J, Jentzsch T, von Rechenberg B, Werner CM (2018). Local effect of zoledronic acid on new bone formation in posterolateral spinal fusion with demineralized bone matrix in a murine model. Arch. Orthop. Trauma Surg..

[64] Bilston LE, Little DG, Smith NC, Williams P, Briody J (2002). Zoledronic acid improves the mechanical properties of normal and healing bone. Clin. Biomech. (Bristol, Avon).

[65] Hughes R (2019). Bone marrow osteoprogenitors are depleted whereas osteoblasts are expanded independent of the osteogenic vasculature in response to zoledronic acid. FASEB J..

[66] Marofi F (2019). Epigenetic mechanisms are behind the regulation of the key genes associated with the osteoblastic differentiation of the mesenchymal stem cells: the role of zoledronic acid on tuning the epigenetic changes. J. Cell Physiol..

[67] Qin L (2006). Multiple bioimaging modalities in evaluation of an experimental osteonecrosis induced by a combination of lipopolysaccharide and methylprednisolone. Bone.

