# Supplementary material for: An injectable self-adaptive polymer as a drug carrier for the treatment of nontraumatic early-stage osteonecrosis of the femoral head
Source: Bone Res. 2022 Mar 12;10:28. doi: 10.1038/s41413-022-00196-y (PMC8918325; doi:10.1038/s41413-022-00196-y)
Supplement: Supplementary file 1 — Supplementary information [file 41413_2022_196_MOESM1_ESM.pdf]

Supplementary materials

An injectable self-adaptive polymer as a drug carrier for the treatment of non-traumatic early-stage osteonecrosis of the femoral head

Ning Kong[a,#], *Hang Yang*[b,#], *Run Tian*[a,#], *Guanzhi Liu*[a], *Yiyang Li*[a], *Xueshan Du*[c], *Huanshuai Guan*[a], *Qilu Wei*[a], *Yutian Lei*[a], *Zhe Li*[a], *Ruomu Cao*[a], *Yiwei Zhao*[a], *Xiaohui Wang*[d], *Kunzheng Wang*[a], *Pei Yang*[a,*]

a. Department of Bone and Joint Surgery, Second Affiliated Hospital of Xi'an Jiaotong University, No. 157 Xiwu Road, Xi'an, 710004, China
b. Harvard John A. Paulson School of Engineering and Applied Sciences, Harvard University, Cambridge, Massachusetts 02138, United States
c. Department of Dermatology, Second Affiliated Hospital of Xi'an Jiaotong University, No. 157 Xiwu Road, Xi'an, 710004, China
d. Department of Spine Surgery, Honghui Hospital of Xi'an Jiaotong University, No.555 Youyi East Road, Xi'an, 710000, China.

[#] These authors contributed equally to this work.

*Corresponding Email: yangpei@xjtu.edu.cn

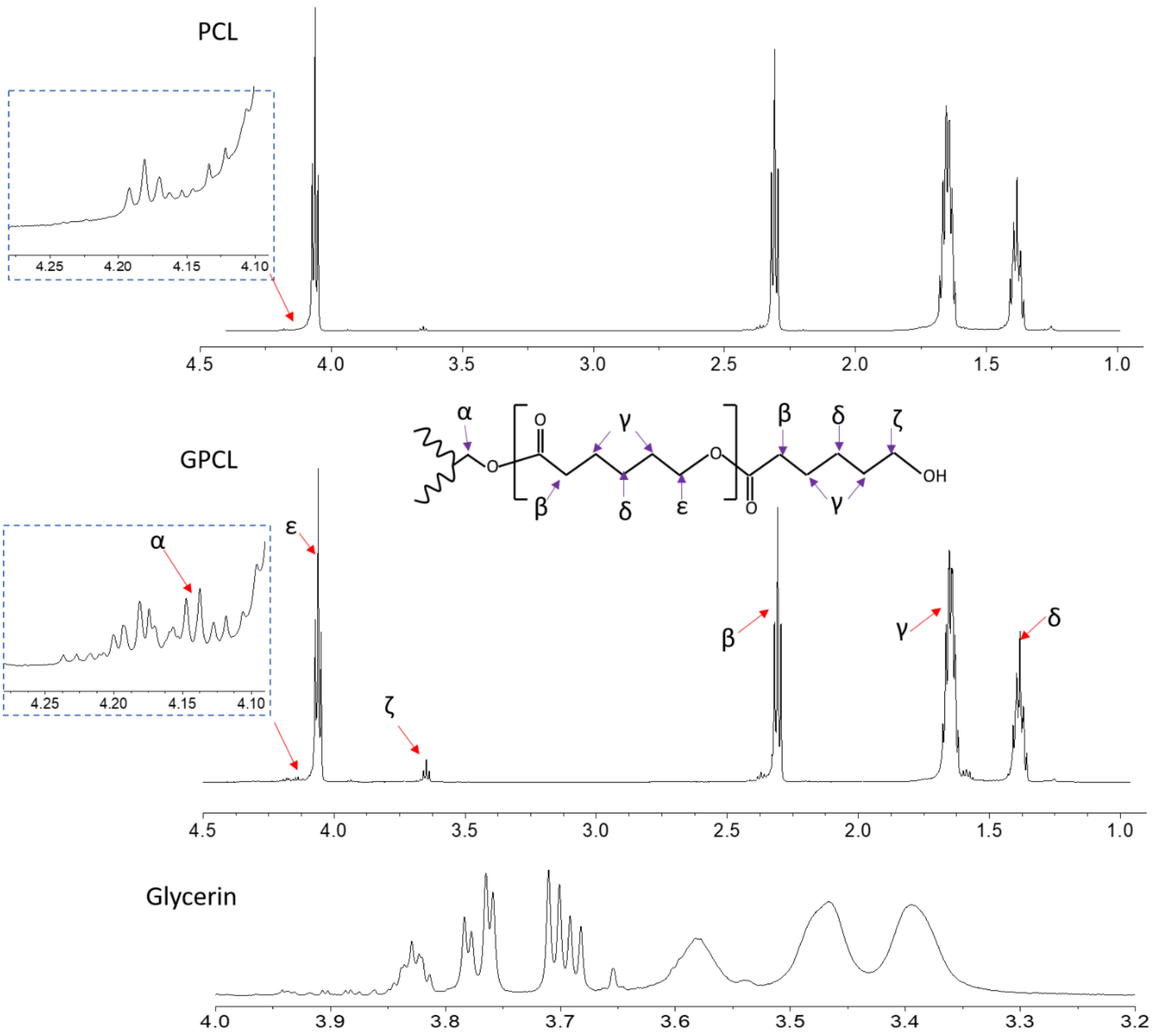

Figure S1. NMR results for the synthesized materials. GPL was obtained by modifying PCL with glycerin. Each NMR graph displays the difference between the PCL and GPCL in high resolution. Letters (α-ζ) represent peaks corresponding to specific hydrogens in the molecular structure.[1,2]

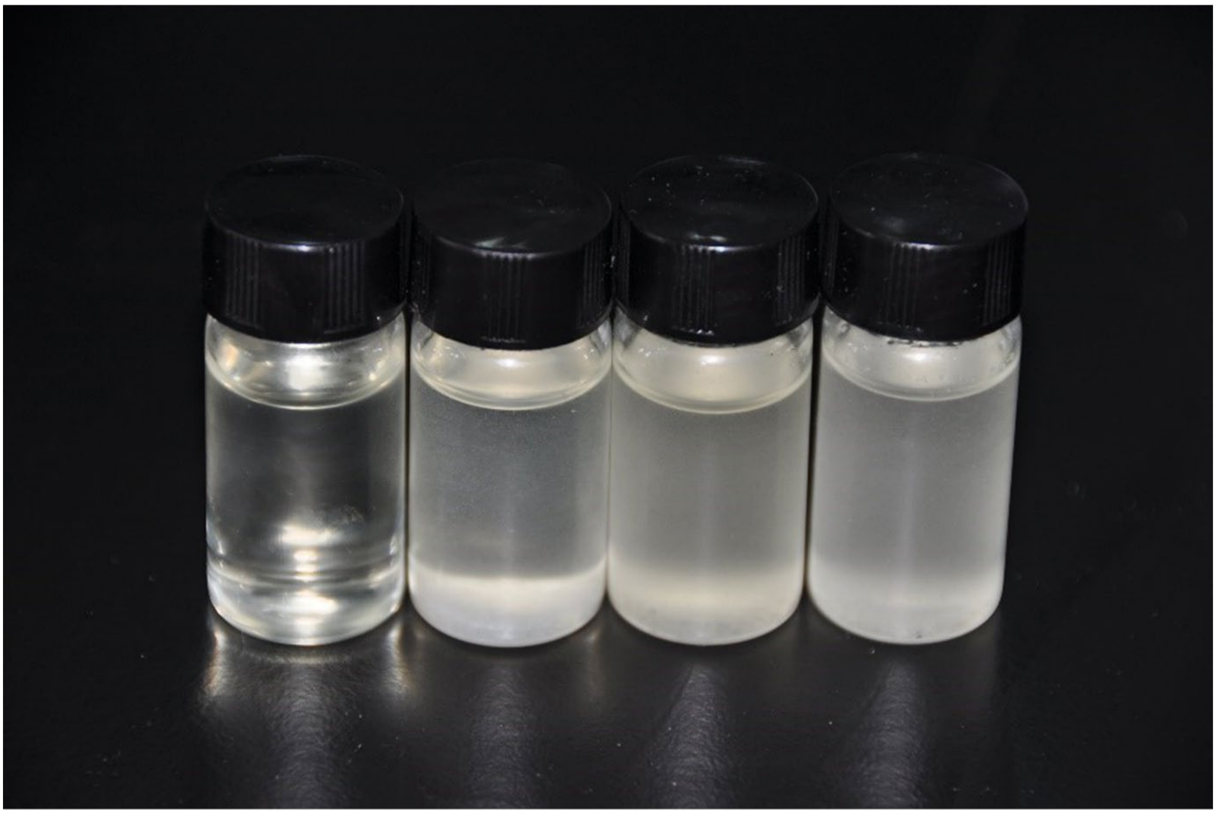

Figure S2. Drug-loaded GPCL. From left to right: GPCL, vancomycin-loaded GPCL, zoledronic acid-loaded GPCL, hydroxyapatite-loaded GPCL. From the images, it is apparent that liquid GPCL alone had a transparent appearance while samples loaded with powder drugs such as vancomycin, zoledronic acid, or hydroxyapatite displayed even dispersion within the liquid GPCL without clotting.

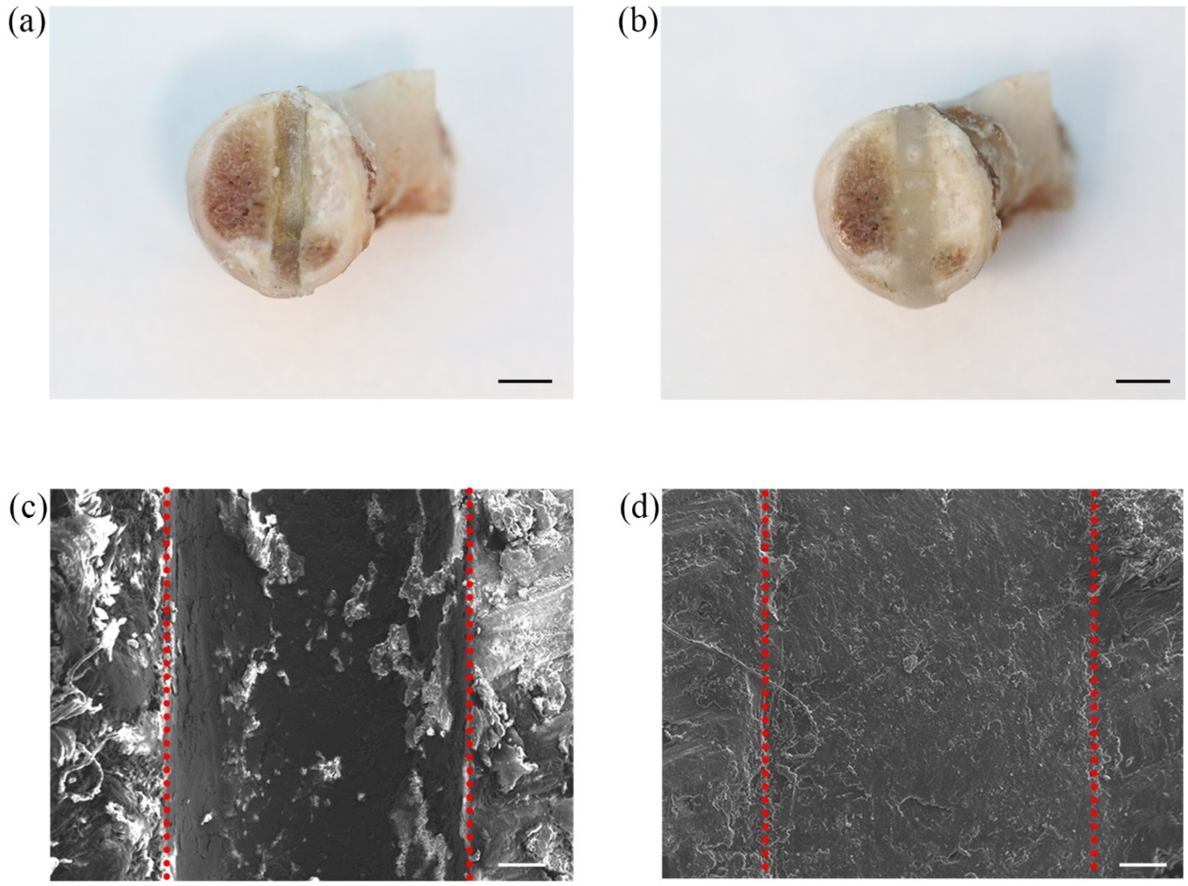

Figure S3. SEM of core decompression channel. (a) General view of empty femoral head CD channel. (b) General view of the GPCL-filled femoral head CD channel. (c) SEM image of an empty femoral head CD channel; (d) SEM image of GPCL-filled femoral head CD channel. Scale bars in (a) and (b) are 3 mm and 300 μm in (c) and (d). From the macroscopic view (Figure S3b) and SEM image (Figure S3d), the injected GPCL fitted well into the channel and filled it tightly.

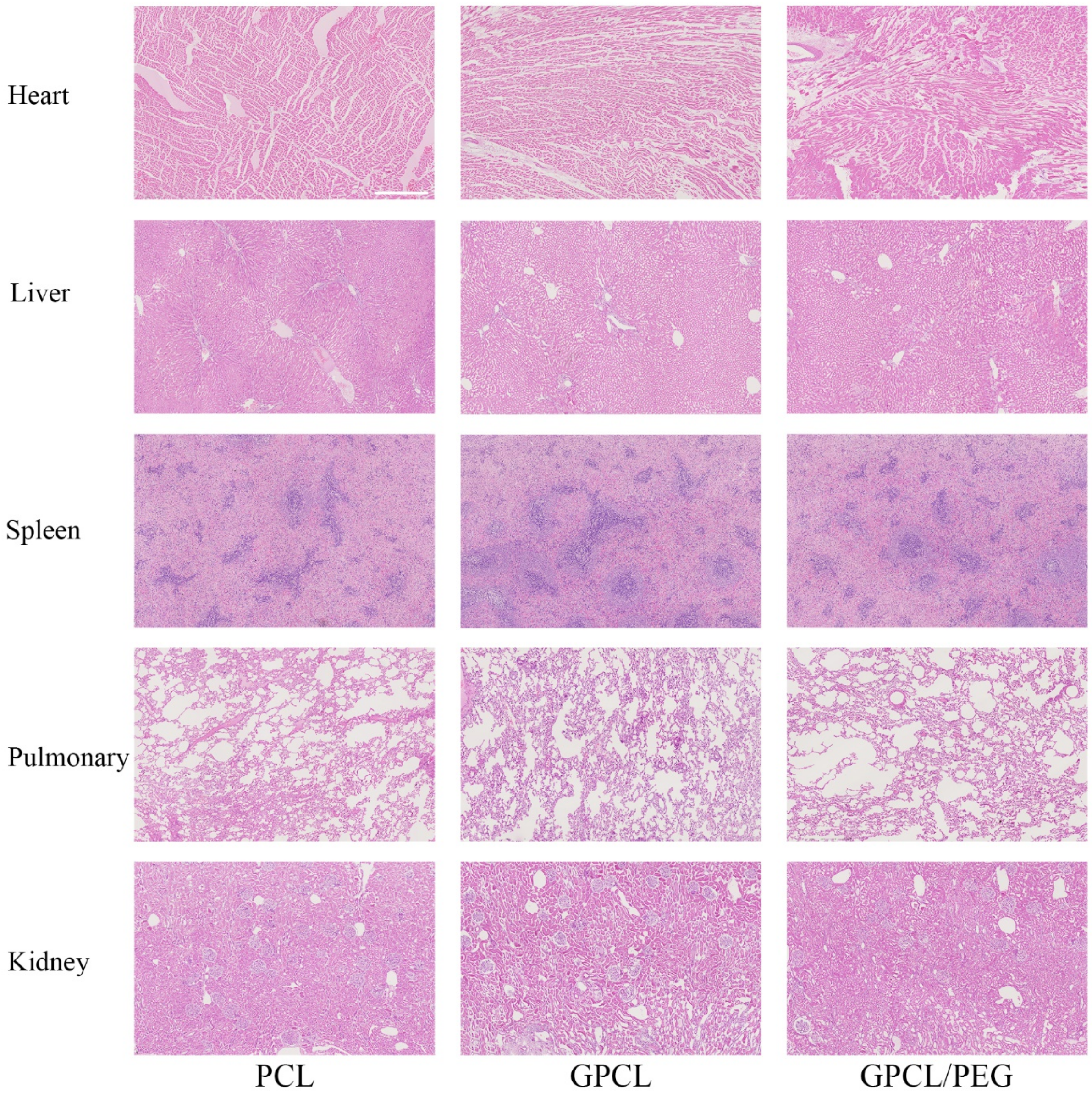

Figure S4. Biocompatibility of implanted materials in organs. HE sections of the main organs, including the heart, liver, spleen, lungs, and kidneys. HE sections of different organs demonstrate that modified GPCL and GPCL/PEG are as safe as FDA-approved PCL for *in vivo* implantation at the organ level. Scale bar: 500 μm. HE stained sections demonstrated that the five main organs maintained normal microscopic structures, and no harmful damage was apparent in those three groups. GPCL, whether alone or in combination with other biocompatible polymers, was found to be safe for *in vivo* implantation at an organ level.

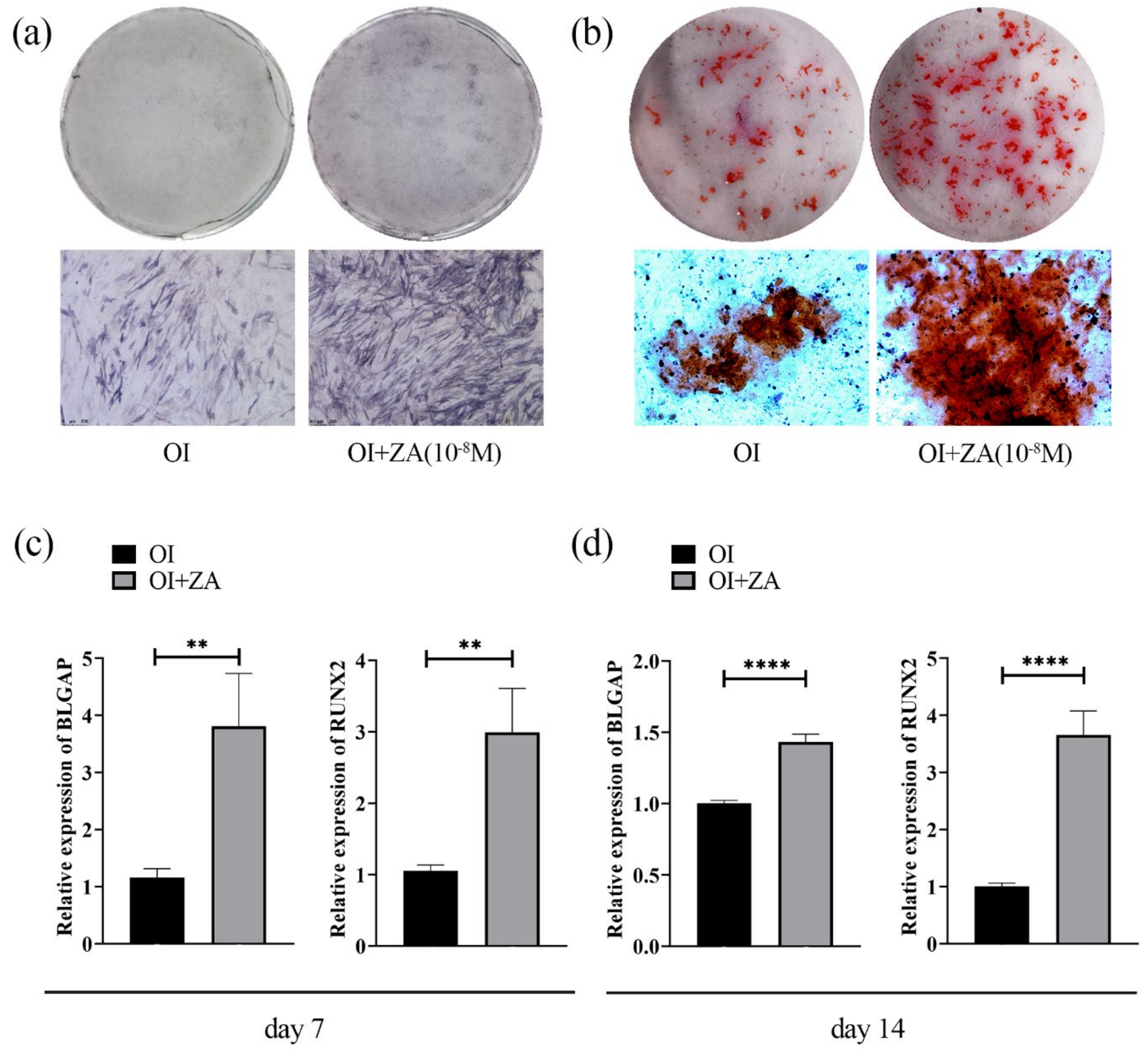

Figure S5. (a) ALP staining at day 7; (b) Alizarin red S staining at day 14; (c) Real-time quantitative PCR of BLAGP(OCN) and RUNX2 at day 7; (d) Real-time quantitative PCR of BLAGP(OCN) and RUNX2 at day 14. On day 7 and day 14, ALP and Alizarin red s staining results showed more alkaline phosphatase formation and calcium deposition respectively after ZA treatment. Real-time quantitative PCR of osteogenetic genes revealed that BLAGP (p=0.0086) and RUNX2 (p=0.0018) were significantly up-regulated at day 7. Similarly, the expression of BLGAP (p<0.0001) and RUNX2(p<0.0001) were significantly promoted at day 14. Primers used in this assay are shown in **Table S1**(n=3).

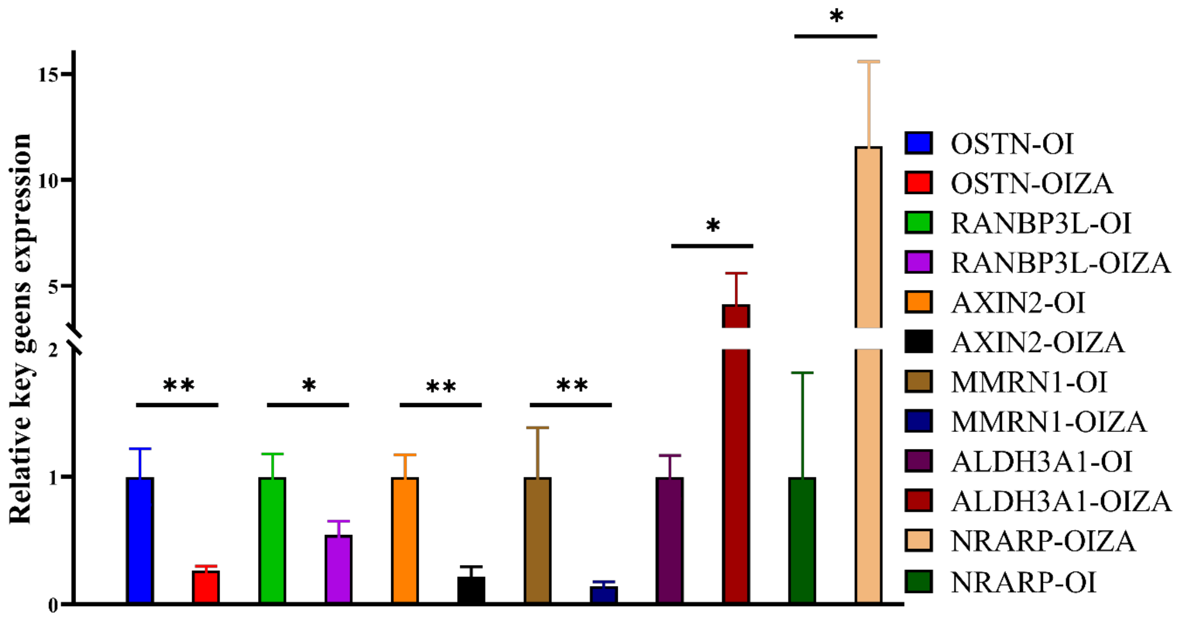

Figure S6. Real-time quantitative PCR of osteoblastic-related DEGs. Negative osteoblastic genes, such as OSTN (0.268±0.033, p=0.005), RANBP3L (0.546±0.109, p=0.0049), AXIN2 (0.222±0.076, p=0.006), and MMRN1 (0.144±0.035, p=0.002) were down-regulated while positive osteoblastic genes, such as ALDH3A1 (4.145±1.455, p=0.017) and NRARP (11.580±4.009 p= 0.018) were up-regulated. Genes expression in the OI group was 1. Primers used in this assay are shown in **Table S1**(n=3).

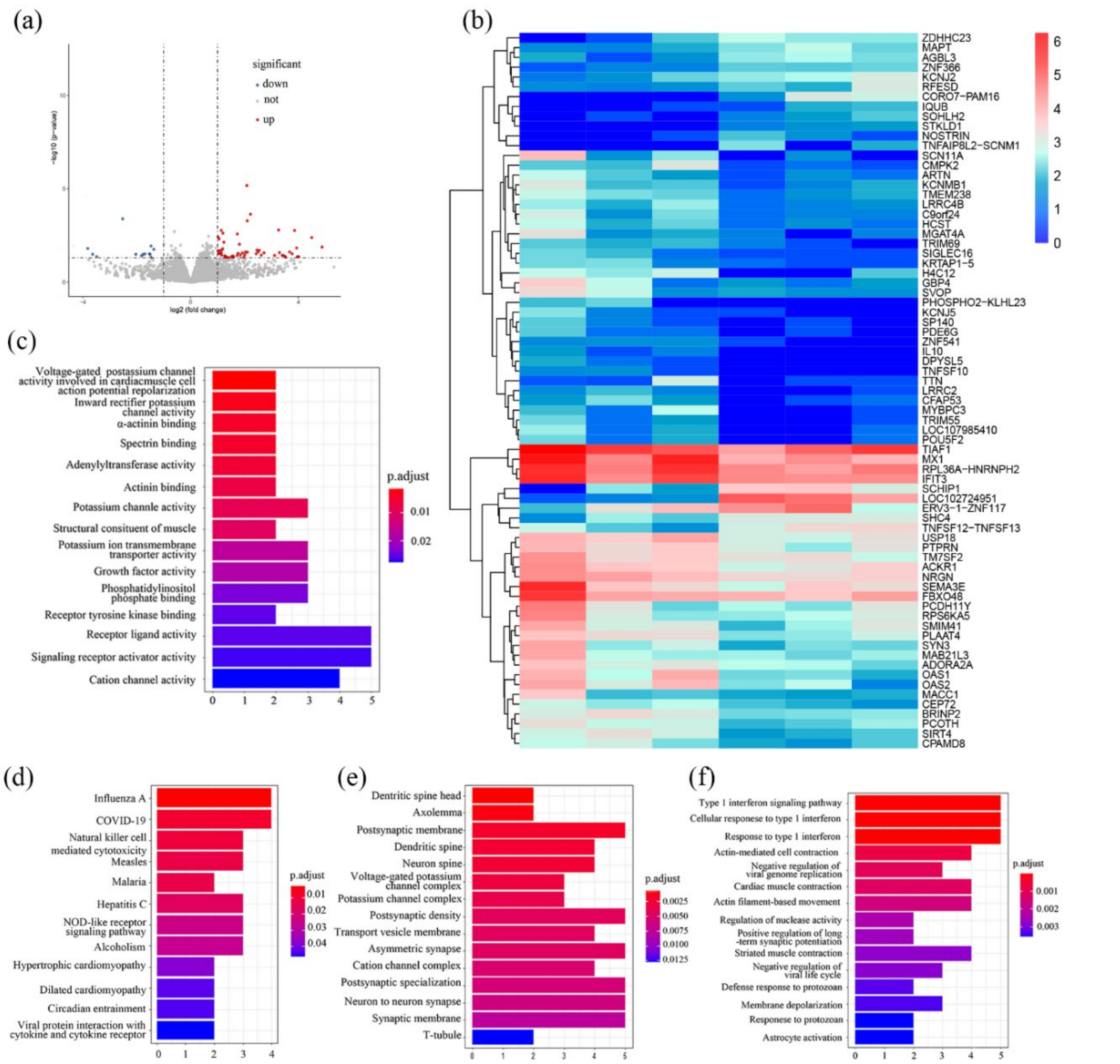

Figure S7. Bioinformatics analysis of the influence of ZA on hMSCs after 7 days *in vitro*. (a) Volcano plot of 16,643 genes from RNA sequencing data. Red points represent upregulated DEGs with $\log_2$foldchange ≥ 1 and $p<0.05$. Blue points represent downregulated DEGs with $\log_2$foldchange ≤ -1 and $p<0.05$. (b) Heatmap of DEG expression levels. (c) GO molecular function analysis of DEGs. (d) GO cell component analysis of DEGs. (e) GO biological process analysis of DEGs (n=3). Bioinformatic analysis demonstrated that there was no clear evidence to predict the osteogenic effects of ZA during the first 7-days of the intervention.

Table. S1

Genes		Primer sequence	Product size
GAPDH	Forward	5'-CAGGAGGCATTGCTGATGAT-3'	120
	Reverse	5'-GAAGGCTGGGGCTCATTT-3'	
RANBP3L	Forward	5'-TCCGTCACTAACCTCCCAACCTTC-3'	123
	Reverse	5'-TTTCACAGGACCTTGTTCAGCACTC-3'	
AXIN2	Forward	5'-CACCACCACCATTCGCAGTACC-3'	131
	Reverse	5'-ACATGCTTCGTCGTCTGCTTGG-3'	
OSTN	Forward	5'-GCAGTCAACACCCACAGTCAGG-3'	82
	Reverse	5'-GGACACCAATTCATCAAGAAGCAAGAG-3'	
MMRN1	Forward	5'-GACTATGCCTTCTGCTTCAGTTCCTC-3'	110
	Reverse	5'-AAGTTCTTGCCTCTGGAGTTGTAGC-3'	
ALDH3A1	Forward	5'-GATGCCGCCACTCGCTACATAG-3'	149
	Reverse	5'-TCACGCTGGTTGATGAACTGGATG-3'	
NRARP	Forward	5'-TCGTGAAGCTGCTGGTCAAGTTC-3'	132
	Reverse	5'-CGTACTTCGCCTTGGTGATGAGATAG-3'	
BGLAP	Forward	5'-CTACCTGTATCAATGGCTGGG-3'	81
	Reverse	5'-GGATTGAGCTCACACACCT-3'	
RUNX2	Forward	5'-AGGCAGTTCCCAAGCATTTCATCC-3'	150
	Reverse	5'-TGGCAGGTAGGTGTGGTAGTGAG-3'	

Reference

1 Shagan, A., Croitoru-Sadger, T., Corem-Salkmon, E. & Mizrahi, B. Near-Infrared Light Induced Phase Transition of Biodegradable Composites for On-Demand Healing and Drug Release. *ACS Appl Mater Interfaces* **10**, 4131-4139, doi:10.1021/acsami.7b17481 (2018).

2 Shagan, A. *et al.* Hot Glue Gun Releasing Biocompatible Tissue Adhesive. *Advanced Functional Materials* **30**, doi:10.1002/adfm.201900998 (2019).
