# Supplementary material for: An injectable self-adaptive polymer as a drug carrier for the treatment of nontraumatic early-stage osteonecrosis of the femoral head
Source: Bone Res. 2022 Mar 12;10:28. doi: 10.1038/s41413-022-00196-y (PMC8918325; doi:10.1038/s41413-022-00196-y)
Supplement: Supplementary file 3 — Animal care [file 41413_2022_196_MOESM3_ESM.pdf]

西安交通大学动物实验伦理审查批准书　　No. XJTULAC2019-1287

Xi'an Jiaotong University Approval for Research Involving Animals

批准日期（App. Date）：2019 年 04 月 19 日　　(YY-MM-DD):2019.4.19

项目主持人姓名<br>Name of Principal Investigator	Pei Yang	申请人单位<br>Department	Second Affiliated Hospital of Xi'an Jiaotong University	
课题名称<br>Project Title	An injectable self-adaptive polymer as a drug carrier for non-traumatic early-stage osteonecrosis of femoral head treatment			
课题来源和批号(Funding Source & Number)	NSFC：81672173			
使用动物情况<br>Animal Requirements	动物来源 Source	Laboratory Animal Center of Xi'an Jiaotong University	品种品系 Species or Strains	SD Rats,<br>New Zealand White Rabbits
	数量 Quantity: Rabbits 50只（♀50只；♂0只） / Rats 40只（♀40只；♂0只）			
	计划开始日期 (Proposed Date of Commencement)	2019.05.01	计划结束日期 (Proposed Date of Completion)	2020.12.31
审查项目<br>Considerations Ethical	1. 该项目的实施是否符合 NIH 和学校动管会相关规定。（Are all procedures to be performed in accordance with the 'Principles of Laboratory Animal Care'(NIH) and guidelines of the laboratory animal care committee of Xi'an Jiaotong University.）<br>2. 所使用的动物品种、等级、规格是否合适，使用数量的计算依据。（The species, strains, grade, specification and number of the animals to be used should be justified.）<br>3. 能否通过改良设计方案替代或减少使用所用动物。(Rational for animal use should be justified, including the alternatives to animal use, a refined study design to replace or reduce animal number to be used..)<br>4. 实验操作中是否善待动物，包括合理的实验终点，麻醉方案，不麻醉的理由及减少相应动物痛苦的措施，实验结束动物的处理，动物安乐死方案等。（Appropriate animal care and handling throughout the experiment, including a scientific sound endpoint; anesthetics, analgesics, sedatives or tranquilizers that are to be used; explanation for any procedure cause unrelieved pain or distress; disposition of animals at end of study; and euthanasia criteria and method.）<br>5. 是否使用对人体或环境有害试剂，有潜在感染性试剂，放射性物质，是否使用遗传修饰试剂，是否进行遗传操作，相应的防护措施。（Are the materials to be used harmful or toxic? Are there any radioactive agents, infectious agents, genetic modified agents, and the genetic manipulation to be used in the experiment? If yes, the safety measures should be specified.）			
实验动物管理委员会意见<br>Comments of the laboratory animal care committee	代表签名：<br>Name of Ethics Committee Representative<br>Date: 2019.4.19			
